# Robust Time Estimation Reconciles Views of the Antiquity of Placental Mammals

**DOI:** 10.1371/journal.pone.0000384

**Published:** 2007-04-18

**Authors:** Yasuhiro Kitazoe, Hirohisa Kishino, Peter J. Waddell, Noriaki Nakajima, Takahisa Okabayashi, Teruaki Watabe, Yoshiyasu Okuhara

**Affiliations:** 1 Center of Medical Information Science, Kochi Medical School, Nankoku, Kochi, Japan; 2 Graduate School of Agricultural and Life Sciences, University of Tokyo, Tokyo, Japan; 3 South Carolina Cancer Center (SCCC), University of South Carolina, Columbia, South Carolina, United States of America; Indiana University, United States of America

## Abstract

**Background:**

Molecular studies have reported divergence times of modern placental orders long before the Cretaceous–Tertiary boundary and far older than paleontological data. However, this discrepancy may not be real, but rather appear because of the violation of implicit assumptions in the estimation procedures, such as non-gradual change of evolutionary rate and failure to correct for convergent evolution.

**Methodology/Principal Findings:**

New procedures for divergence-time estimation robust to abrupt changes in the rate of molecular evolution are described. We used a variant of the multidimensional vector space (MVS) procedure to take account of possible convergent evolution. Numerical simulations of abrupt rate change and convergent evolution showed good performance of the new procedures in contrast to current methods. Application to complete mitochondrial genomes identified marked rate accelerations and decelerations, which are not obtained with current methods. The root of placental mammals is estimated to be ∼18 million years more recent than when assuming a log Brownian motion model. Correcting the pairwise distances for convergent evolution using MVS lowers the age of the root about another 20 million years compared to using standard maximum likelihood tree branch lengths. These two procedures combined revise the root time of placental mammals from around 122 million years ago to close to 84 million years ago. As a result, the estimated distribution of molecular divergence times is broadly consistent with quantitative analysis of the North American fossil record and traditional morphological views.

**Conclusions/Significance:**

By including the dual effects of abrupt rate change and directly accounting for convergent evolution at the molecular level, these estimates provide congruence between the molecular results, paleontological analyses and morphological expectations. The programs developed here are provided along with sample data that reproduce the results of this study and are especially applicable studies using genome-scale sequence lengths.

## Introduction

Despite great progress over the past decade, the evolutionary history of placental mammals remains controversial. While a consensus is emerging on the topology of the evolutionary tree [1]–[5], although with occasional disagreement [6]–[8], divergence times remain uncertain. The age of earlier nodes and in particular the root, remain especially uncertain in the absence of definitive placental fossils deeper into the Cretaceous [4]–[6], [9]. Both paleontological and morphological studies suggest that the radiation of placental orders and super orders occurred close to the Cretaceous–Tertiary (K–T) boundary about 65 million years ago (mya) [9], [10].

In contrast, molecular studies have suggested markedly older origins for many superordinal groups and that some extant orders diversified before the K–T boundary. The root of living placental mammals has been reported to be in the range of 100–140 mya [4], [11]–[15] even with application of rate-adjustment techniques [4], [15]. The molecular consensus of an old root is becoming strong enough that it may become dogma without a close examination of the assumptions being made in such analyses. The true age of the orders and superorders has important implications for determining the overall paleoecology and biogeography of placental mammals during a period that included the breakup of continents and the extinction of terrestrial dinosaurs. Reconciling the different paleontological and molecular divergence time estimates has important implications for basic methodologies that are central to evolutionary study.

The strength of molecular divergence time studies is their potential to draw information from very long aligned sequences of many species. It is widely assumed that a huge amount of sequence data and the approximate rate constancy of sequence evolution [16] make molecular estimates more reliable than those based solely on fossil data. Such analyses on a genomic scale are generally anticipated to be decisive because they are expected to be free of stochastic noise [17], [18].

However, molecular studies acknowledge the problem of misspecification of the model of sequence change, which may result in seriously biased estimation. The relationships among placental orders do vary according to the data used and the taxa sampled [1]–[8], [13]–[15], [19]–[22]. Most methods of phylogenetic inference rely strongly on probabilistic models of sequence evolution, and neither directly detect nor correct for convergent evolution [23]. When left uncorrected, such “homoplasy” may attract lineages and underestimate certain pairwise distances. This in turn distorts branch (edge) length estimates, which are of primary importance for divergence time estimation. While some methods will detect potential convergent evolution, for example Hadamard conjugations and SplitsTree [24], Kitazoe et al. have developed multidimensional vector space (MVS) representation methods to both detect convergent evolution among estimated distances and to also correct for this bias [25]–[27].

A further problem facing molecular dating is the evolutionary rate constancy, or lack of constancy, over long periods. Since its proposal in 1965 [28], the molecular clock hypothesis has been one of the most hotly debated subjects in evolutionary biology. It is now widely accepted that a molecular clock tends to hold well for closely related organisms, but breaks down with increasing evolutionary divergence. To adjust for the inevitable fluctuation of evolutionary rates, nonparametric [17], [18], local clock [29], [30], and hierarchical Bayesian [31] methods have been developed. These methods are robust against stochastic fluctuations of evolutionary rates [31]–[33]. However, they may cause serious problems when pronounced transient changes of rate occur, because they overly smooth such rate changes. In this article, we show the magnitude of such problems using clear worked examples.

To improve the detection of, and robustness to, abrupt rate changes, we have developed a new procedure that minimizes the local variability of the inverse of the evolutionary rate. Just as the effective size of fluctuating populations is represented by the harmonic mean over time, the mean evolutionary rate among lineages is expressed better by the harmonic mean. This approach is especially useful when branch lengths measured in the expected number of substitutions per site (the products of rates and times) are estimated accurately, and there are either rapid transient changes of rate (hence large rate heterogeneity) or a general bias towards a speed up or slow down in rates through time.

Using this new procedure, an analysis of 69 mitochondrial protein sequences (3660 amino acid sites in total) from placental mammals identified a rapid acceleration of evolutionary rate for the lineage directly leading to the common ancestor of Supraprimates and an even more marked one for the lineage leading to Laurasiatheria. This acceleration was followed closely by a strong deceleration, which persisted in nearly all lineages of Laurasiatheria. In contrast, almost all lineages of Afrotheria and Xenarthra seem to have retained rates similar to that of the root. This view is in marked contrast to current rate-change penalty functions. The robustness of the new procedure is assessed using simulations that show the types of change that most concern biologists; speedups or slowdowns through time, transient rate changes, and rate changes that are do not follow a normal or transformed normal distribution, as well as stochastic variation. A revised estimate of the origin of placental mammals is as young as 84 mya, which is much more recent than current estimates using molecular data. The inferred age of deeper splits in the placental tree are compared with the rate of occurrence of new species from the North American fossil record. These two sources of data are far more congruent than is suggested by using current, possibly strongly misleading, dating methods.

## Results

### Revised rates and times of mitochondrial protein sequences from placental mammals

Mitochondrial protein sequences are used widely in phylogenetic studies and have been particularly popular in studying placental mammals. A desirable feature of these data is relatively long sequences, good taxon sampling and very little missing data. Following alignment, we retained 3660 amino acid sites present in all of 62 placental mammals plus seven outgroup taxa (Table S1 gives the accession numbers of protein sequences). In contrast, large nuclear protein datasets of mammals with diverse taxon sampling [e.g.3], [4] show a large proportion of missing data. Such missing heterogeneous data may lead to complex systematic errors in distance estimation [34]. This is particularly relevant since MVS analyses work best with low stochastic noise (hence relatively long sequences), diverse taxon sampling with a considerable proportion of taxa showing minimal convergent evolution, and minimal bias of the input distances from sources such as missing data.

We adopted a standard two-step procedure to estimate divergence times. The first step is to estimate the phylogenetic tree with unconstrained branch lengths in units of expected numbers of substitutions per site. Given the problems with convergent evolution in mitochondrial data [4], [5], [22], [25], we used the MVS procedure to correct the input distances for convergent evolution. A variant of the core set approach [27] to MVS was used in this instance (Materials and Methods). Fixing the topology to the tree obtained by MVS, which is very similar to trees obtained by previous authors using varied data sets [2]–[5], branch lengths were reestimated using maximum likelihood (ML) in the program PAML [35]. We used the JTT [36]+Gamma model with α = 0.5 for these analyses. The second step uses the tree and its branch lengths, some fossil constrained nodes, and a penalty or cost function to dampen rate changes, to infer divergence times of all nodes and evolutionary rates along all branches.

Three cost functions, F_ADD_, F_LOG_, and F_IR_, were applied to the MVS and ML trees. These functions penalize the fluctuation of rates, and do this on either a linear scale (here called the ADD function), on the log rate (LOG), or on the inverse rates (IR), respectively (see equations (1), (2), and (4) in Materials and Methods). The tree estimated by MVS and F_ADD _is denoted MVS-F_ADD_ and so on (while ML-F_ADD_ indicates use of ML branch lengths).

**Figure 1:**
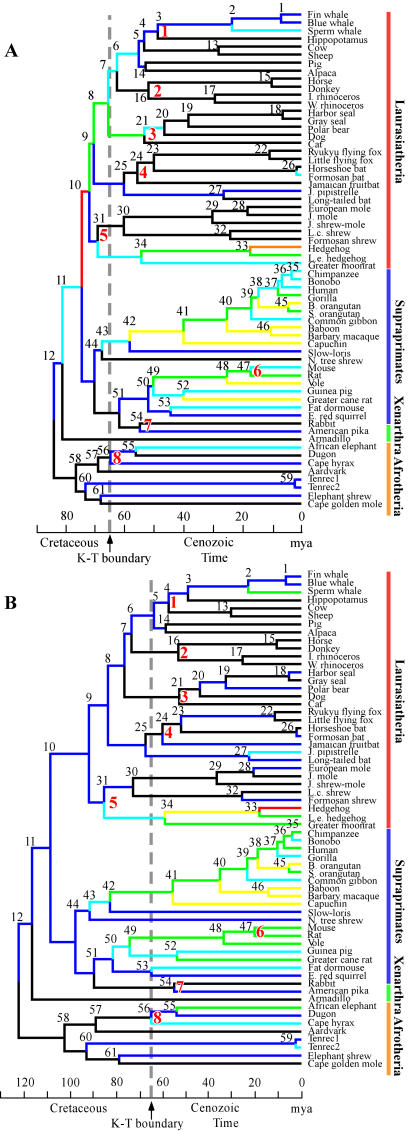
Molecular divergence times and evolutionary rates of placental mammals. A and B show the MVS-F_IR_ and ML-F_LOG_ trees, respectively. The numbers 1–61 denote the ancestral nodes. The red numbers 1–8 indicate the internal nodes with fossil constraints which are as follows: 1, 49–61; 2, 52–58; 3, 45–63; 4, 43–60; 5, <63; 6, >12; 7, 36–55; 8, 54–65 (all in mya) [see 4], [15]. The colors of the branches denote inferred evolutionary rates (in units of ×10^−9^/site per year) as follows: black, <0.2; dark blue, 0.2–0.3; light blue, 0.3–0.4; green, 0.4–0.5; brown, 0.5–0.6; yellow, 0.6–0.7; and red, >0.7.

To calibrate these trees, we used eight fossil constraints, all taken from previous studies (see Figure 1 caption) [4], [15]. The divergence times were estimated by then minimizing each cost function subject to these constraints (see Materials and Methods). The CI was calculated using a likelihood interpretation (see Materials and Methods) of the cost function residual analogous to the method used in Multidivtime [31]. This captures the error caused by deviations of the tree's branch lengths away from their expected values and includes the unpredictability of rate changes, which might mimic Brownian motion, for example. It will also incorporate variability arising from ancestral polymorphism. Polymorphism will cause fluctuations of the edge lengths of the tree when the analysis has multiple nodes constrained by fossil or other data [c.f. 13]. Generally, the time at the most recent common ancestor of sequences from different species is older than the time at speciation. The extent of this difference varies among internal nodes due to both the stochastic nature of the coalescent and factors governing its expected magnitude, such as population size. The cost function does not take account of this bias, but the CI does include the variance arising from ancestral polymorphism at internal nodes.

The MVS-F_IR_ analysis (Figure 1A) estimated the age of the root at 84.2 mya with a 95% confidence interval (CI) of 80.7–88.4 mya (Table 1). This time is much more recent than the result of the ML-F_L_ analysis (Figure 1B), i.e., 122.2 (95% CI, 112.2–144.7) mya. Note that, ML-F_L_ is giving results consistent with that reported in earlier studies [1]–[5], [12]–[15], indicating that the younger root for placentals returned using the function F_IR_ (see below) is due to the method and not the data. The difference between the F_LOG_ and the F'_LOG_ function in table 1 is explained in Materials and Methods. It is the F_LOG_ method that most closely approximates the Brownian motion assumed by Multidivtime.

**Table 1:** The age of the root of placental mammals

Tree model	Cost function					
	F_IR_	F_ADD_	F_LOG_	F'_LOG_	F_ADD_(r8s)	F'_LOG_(r8s)
MVS	84.2(80.7, 88.4)	Infinity	105.0(97.0,117.0)	91.8(84.5,104.7)	160.5	91.1
ML	106.4(102.4,110.6)	Infinity	122.2(112.2,144.7)	112.0(102.0,121.2)	122.2	111.6

The age of the root using MVS and ML branch lengths was estimated after minimizing various penalty functions with the same fossil constraints. All times are in mya, and 95% confidence intervals were estimated by the sum-of-squares method described in Materials and Methods. The times estimated by the *r8s* program use cross-validation and penalized likelihood methods with essentially the same penalty functions [18], but do not report comprehensive errors.

**Figure 2:**
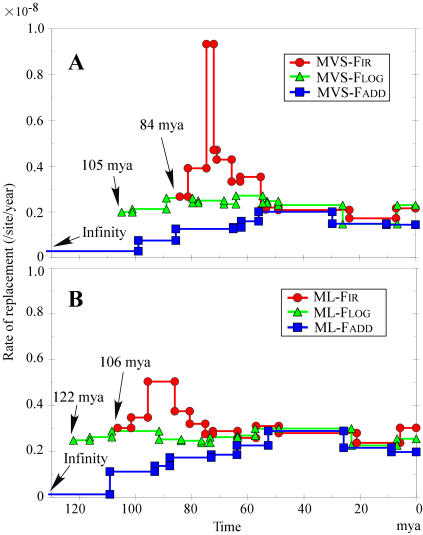
Evolutionary rate changes from the root to the fin whale. Figures A and B, respectively, trace the estimated rates along edges on analyses using MVS and ML branch lengths. The root time of of the F_ADD_ analysis in Figure A and B was set at a large value (400 mya) because the numerical calculation continues towards an infinite root time.

Figure 1 shows the estimated ancestral rates across the tree, while Figure 2 represents the inferred evolutionary rates going from the root to a terminal. The single instance of root to fin whale is shown in figure 2, while figure S1 traces the evolutionary rates inferred by different methods along seven representative lineages. The MVS-F_IR_ tree identified an abrupt acceleration of evolutionary rate near the common ancestor of both Supraprimates and Laurasiatheria, then a very strong acceleration in just the ancestral lineage of Laurasiatheria (Figures 1A and 2A). This acceleration was followed closely by a strong deceleration along nearly all lineages of Laurasiatheria. In contrast, almost all lineages of Afrotheria and Xenarthra have retained rates close to that of the root. There are also sporadic later accelerations, for example that among hedgehogs and Moon rat (Figure 1A).

The cost function F_IR_ detected an acceleration–deceleration pattern near the base of Laurasiatheria using both the MVS and ML branch lengths (the red lines of Figure 2), whereas the function F_LOG_ showed a far more flat prediction of generally lower evolutionary rates that led to older root times (the green lines of Figure 2). Even more extreme, the cost function F_ADD_ inferred gradually decreasing rates in all deep branches of the MVS and ML trees (the blue lines of Figure 2), and the root time tended to infinity. Table 1 summarizes the root time values estimated by various models (all the node divergence times from these models are listed in Table S2).

Table 1 also compares the inferred times with those obtained from *r8s*, a popular program for estimating absolute rates [17], [18]. This program has two options of for its cost functions, F_ADD_ and F′_LOG_ (equation (3) in Materials and Methods). It also takes account of stochastic noise caused by the finite length of the sequences used to derive the branch lengths using a penalized-likelihood approach. The fitting to the estimated branch lengths is expressed by the log likelihood, while the weight for the cost function is estimated using cross validation. In our mitochondrial dataset, with its long sequences, the root time values changed little due to the cross validation effect (the reason is explained in Materials and Methods). Further, root divergence time estimates returned by *r8s* do not show confidence intervals in table 1, since *r8s* only assesses the sampling variance of node times due to stochastic errors in branch lengths due to finite sequence length (it does this via the bootstrap) and does not include the effect of stochastic variation of evolutionary rate. This source of error remains as sequence lengths go to infinity, and will be dominant with long sequences.

### Agreement with fossil data

**Figure 3:**
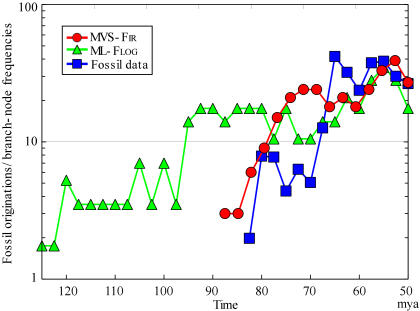
Comparing molecular and fossil records of placental diversification. The blue squares show the rate of the appearance of new species based on the fossil record [adapted from 3]. At present, such quantitative data are limited to the well-studied North American record. The red circles represent the chronological distribution of node density (or splits) on the MVS-F_IR_ tree (Figure 1A). The green triangles represent split frequency on the ML-F_LOG_ tree (Figure 1B). The node density is given by the number of nodes in an 8 myr sliding window. A scaling constant for the molecular frequencies was calculated via a least-squares fit to the fossil data in the period 85–50 mya.

Figure 3 represents the chronological distribution of the internal node density for the MVS-F_IR_ tree (Figure 1A) compared with the rate of new species appearing in the well-studied North American fossil record. To avoid potential bias of divergence times in the molecular tree due to species sampling, the scaling constant was determined by a least-squares fit to the fossil data in the period 85–50 mya.

The MVS-F_IR_ analysis reconciles molecular with fossil data in two ways. First, the chronological distribution of the internal node density is clearly largely consistent with the rate of appearance of novel fossil species. This is despite the fact that the paleontological data assessed are limited to the well-studied North American record [10]. Both the MVS-F_IR_ tree and the fossil record suggest accelerating taxonomic diversity near the K–T boundary, rather than a longer slower buildup [2]–[5], [12]–[15]. Use of the less robust F_LOG_ criterion seems to suggest a prolonged increase in diversity that agrees far less with the quantitative fossil record. This congruence with fossils does not automatically show that the combination of MVS and F_IR_ is the best way to analyze this data, but it does reframe the discussion of the relationship between the fossil and molecular times into one where the molecular dates are being looked at far more critically.

Second, and more indicatively, the cost function F_IR_ resolves incongruence among the fossil constraints/inferred divergence times in different parts of the molecular tree. The best fossil constraints in Laurasiatheria suggest much older times than constraints in other parts of the tree [4], [37]. The MVS-F_IR_, MVS-F_LOG_, and MVS-F_ADD_ trees with all constraints give the root as 84.2, 105.0, and ∞ mya, respectively (Table 1). Removing the whale–hippo constraint gives 82.4, ∞, and 91.2 mya; removing the horse–rhino constraint gives, 82.8, 105.0, and ∞ mya; and removing both sets of constraints gave 82.9, 87.2, and ∞ mya. Thus, in this case only the F_IR_ tree is insensitive to “constraint sampling”.

Finally, there is a good fossil calibration for tarsier [4] that was withheld because it is not used in both references 4 and 15. It suggests that human and tarsier split around 50–60 mya and the molecular trees suggest the human-loris split was not much earlier (probably <5 myr earlier). Despite our use of whale–hippo and horse–rhino calibrations, the MVS-F_IR_ tree (Figure 1) gives a human-loris split close to this age, in contrast to earlier studies [4], [15], [33].

### Sensitivity and robustness of F_IR_ assessed by simulations

We highlight two distinct properties of the new function F_IR_ with the help of evolutionary simulations and worked examples. The first property is its ability to detect a transient acceleration of evolutionary rate. Such an effect might be caused by a burst of positive selection and/or a bottleneck in population size. The second is to assess the effects of both stochastic fluctuations and systematic bias on the robustness of estimated times. Here, we model bias in the form of either a general slowdown or a general acceleration of evolutionary rate across the whole tree.

**Figure 4:**
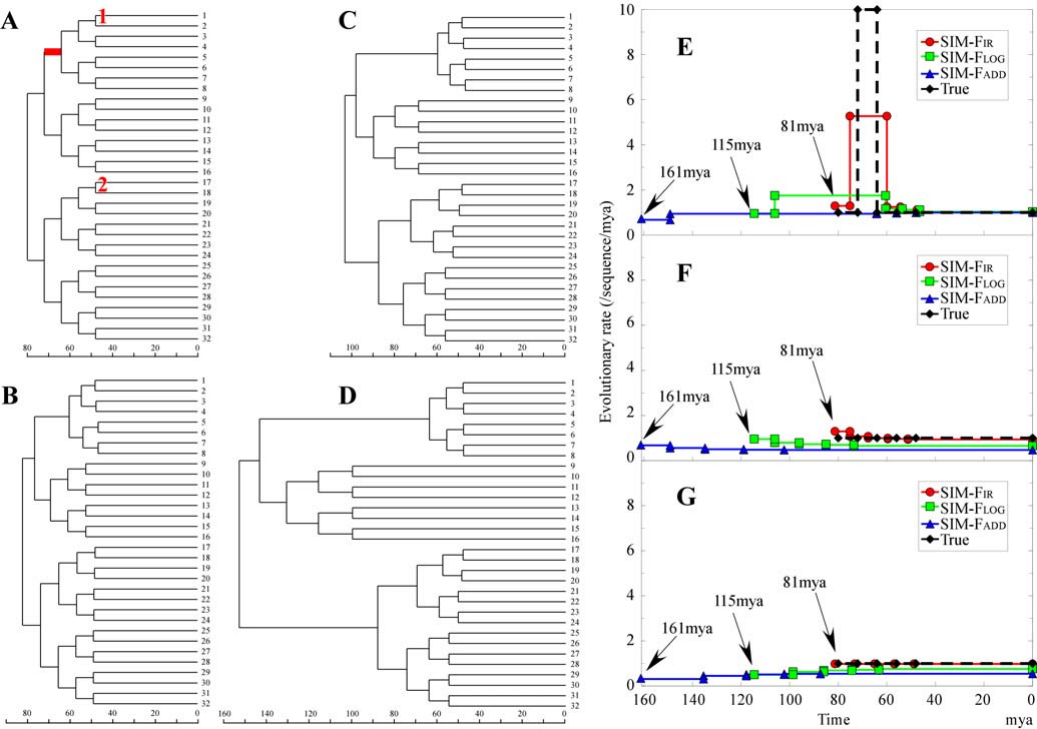
A worked example of the effect of a transient elevation of evolutionary rate upon estimated divergence times. In this worked example using a symmetric 32-taxon tree (Figure A), a global molecular clock holds, except for a short-term increase in evolutionary rate along one branch (the red line in Figure A). The true root time was set to 80 mya, and the times at the internal nodes are 48, 56, 64, and 72 mya. The deep internal branch (the red line in Figure A) is given an evolutionary rate ten times that of the remaining edges. The various cost functions were minimized subject to two calibrated nodes (the red numbers 1 and 2 in Figure A), using the exact branch lengths of this example as input data. The cost functions F_IR_, F_LOG_, and F_ADD_ inferred the weighted trees of Figures B, C and D, respectively. Figures E–G show the trace of evolutionary rates along the lineages from the root to taxa numbers 5, 9, and 25 of Figure A, respectively. The inferred age of the root for each cost function is shown with an arrow. The function F_IR_ recovered the original pattern of rate change, whereas the other two functions inferred far more gradual changes, which resulted in a substantial overestimation of the root time.

We first modeled a strong instantaneous acceleration as an analogue to what is inferred by F_IR_ to have occurred ancestral branch leading to Laurasiatheria. We simulated a 32-taxon symmetric tree in which a molecular clock holds except for an abrupt elevation (by a factor of 10) of evolutionary rate along a short internal branch (the red line in Figure 4A). This example is useful for demonstrating the efficacy of the F_IR_ function and something similar appears on both the MVS-F_IR_ and ML-F_IR_ mitochondrial trees. The times at the internal nodes were set to 48, 56, 64, and 72 mya, and the root time was set to 80 mya. On this weighted tree, the cost functions were minimized with two constrained node times, which corresponded to two fossil calibration points. Only the F_IR_ function accurately estimated the true divergence times and appeared robust against the abrupt change (Figure 4B). In contrast, the functions F_LOG_ and F_ADD_ inferred gradual rate changes (Figure 4E), which are erroneous and lead to overestimation of the root time along with that of many other nodes (Figures 4C and 4D). Table 2 summarizes the root time values inferred by various models. Table 2 includes the result of *r8s* with the cross-validation method.

**Table 2:** Estimated age of the root in the presence of an abrupt acceleration then deceleration of ancestral rates

True value	Cost function					
	F_IR_	F_ADD_	F_LOG_	F'_LOG_	F_ADD_(r8s)	F'_LOG_(r8s)
80	81.3	161.1	114.5	101.7	160.5	108.7

These estimates are from a worked example with an abrupt acceleration then deceleration (the red line of Figure 4A) which occurs deeper in the tree than the calibration points. The true root age is 80.0 mya. The estimated values are made by minimizing the cost functions (that also appear in table 1), but upon the weighted constrained tree of figure 4A.

We next simulated stochastic rate fluctuations by themselves, plus either a prevailing slowing down or acceleration of rates through time. Such simulations are distinct from a Brownian-type process, and are used to gauge the general robustness of the functions. To impose rate fluctuations on the same basic tree as Figure 4A, but without the abrupt rate change, the age of the root was set to 100 mya. We assumed infinite length of sequences and that the branch lengths are known without uncertainty. Next a branch was randomly selected proportional to its duration in time and all its descendant branch lengths were multiplied by a factor (chosen randomly) from a uniform distribution of range 0.5–1.5. A total of 25 such rate changes were placed on the tree to give the final branch lengths for that tree. These branch lengths were used as the input data and the various cost functions were minimized with on calibrated internal node, and then determined all node times. We repeated this procedure 600 times to obtain the mean and standard error of the root time. We also simulated the two other cases. The only difference was the range of the uniform distribution used. Using a range of 1.0–1.75 the model is biased toward rate acceleration through time, while using and 0.25–1 induces a rate slow down through time (a probable situation in the placentals generally). As table 3 shows, the function F_IR_ gave reasonable estimates with a bias towards deceleration, whereas F_ADD_ gave an infinite root time in 139 of 600 samples. These undefined root-time values were set arbitrarily to 200 mya before calculating the mean and standard error.

**Table 3:** The age of the root inferred by functions F_IR_, F_LOG_, and F_ADD_ on simulated data with random auto-correlated rate changes

Rate range	Cost function		
	F_IR_	F_LOG_	F_ADD_
0.25–1.0	111.3±11.1	125.1±12.7	172.0±33.9
0.5–1.5	100.7±4.4	103.3±3.9	103.6±5.1
1.0–1.75	93.2±2.0	94.0±2.1	94.3±2.4

In these simulations, the length of a branch (on what tree!) is selected randomly proportional to its true duration in time. A rate adjustment (change) factor is then chosen randomly form a uniform distribution and all its descendant branch lengths were then multiplied by this factor. A total of 25 such random rate changes were placed on the tree, then the branch lengths (measured in the product of rate and time) of the weighted tree were passed on to the time estimation procedures. The whole procedure was repeated 600 times to obtain the average root time and standard error. Three different ranges were used for the uniform distribution of rate changes. The first has a range of 0.25 to 1 and represent a strong persistent bias towards rate deceleration. A range of 0.5 to 1.5 gives minimal rate change bias, but retains stochastic fluctuations. Finally a strong acceleration effect is achieved by the use of the range 1.0–1.75. The function F_ADD_ gave an age of the root tending to infinity on 139 of the weighted trees simulated under the deceleration model. In such cases the root time is set to 200 mya in order to allow the mean and standard deviation of this cost function to be calculated.

### Reduced bias in branch length estimation by the MVS model

**Figure 5:**
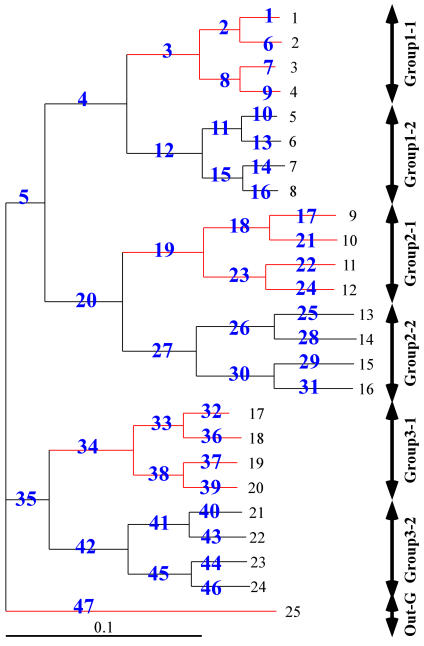
A simulation of convergent evolution. Amino acid substitutions were evolved on the shown weighted tree using the JTT model [36] of amino acid substitutions. After this, 30% of sites were swapped between the four lineages indicated by the red lines.

The MVS model was shown previously to recover the correct tree in a simulation with two strongly convergent lineages [26] that were grouped erroneously by standard methods (including the neighbor joining (NJ) [38] and ML [35] methods). Here, we examined a different question; this is how well the MVS method recovers the true branch lengths, which are of primary importance when estimating divergence times. We simulated the evolution of a sequence of 10^4^ amino acid sites, following the tree depicted in Figure 5. The model of amino acid substitutions used was the JTT [36]. Convergent evolution was then imposed on this data. This was done by sharing parts of the sequences among three ingroup clades plus the outgroup. The red lines in Figure 5 indicate which lineages shared sequence and were therefore subject to a form of convergent evolution.

Pairwise distances were then estimated from the terminal sequences obtained above using the same JTT model. A modified MVS core-set procedure [27] was able to recognize that the tree consisted of three groups of eight sequences which had additive distances within each group and the outgroup. The MVS procedure then converted the distances within the three ingroups into three sets of perfectly additive distances and sequentially combined them to form a single core set (Materials and Methods). The final MVS tree was obtained by modifying the distances between this single ingroup core set and the outgroup following the rules described in Materials and Methods.

**Figure 6:**
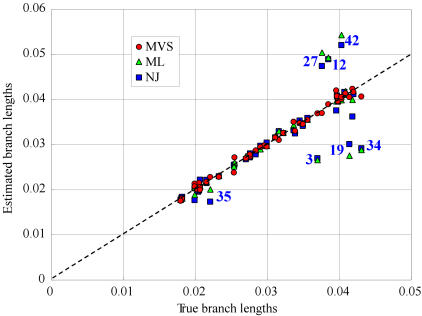
Branch lengths estimated by MVS, NJ, and ML for the example of convergent evolution in figure 5. The tree topology inferred by these methods was identical to the tree that generated the data, so the estimated branch lengths are plotted against their true values. The blue numbers show the branch index, as used on Figure 5, of outliers. Note, all the outliers are internal branches ancestral to the four lineages undergoing convergent evolution or are ancestral to the sister group to these lineages. Branch lengths ancestral to the groups undergoing convergent evolution are underestimated by the ML and NJ methods, whereas those ancestral to their sister taxa are overestimated.

The branch lengths recovered by the MVS model reproduced the true values accurately (Figure 6). The distribution around the diagonal line in Figure 6 represents the stochastic fluctuation of the estimated distances; that is, the magnitude of this fluctuation did not change after the simulation was rerun without convergent evolution. In contrast, the NJ and ML trees returned branch lengths that were affected clearly by convergent evolution. The worst affected branch lengths were ancestral to the groups showing convergent evolution (underestimated) or else were leading to the sister groups of the groups affected by convergent evolution (overestimated).

## Discussion

**Figure 7:**
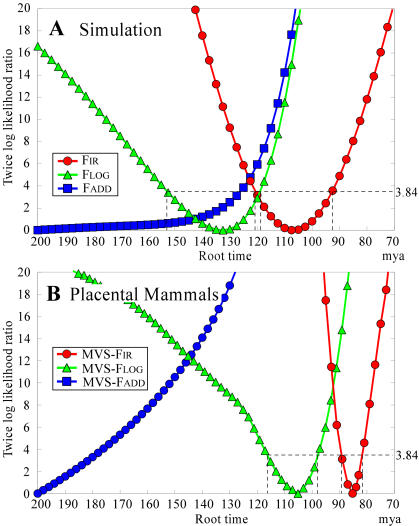
Log-likelihood profiles of the F_IR_, F_LOG_, and F_ADD_ cost functions about the estimated ages of the root. This figure shows the profile of the cost function with respect to the age of the root estimated by three different cost functions. Because the root age estimated by function F_ADD_ was going to infinity, it was set to 200 mya for illustrative purposes. Figure A is a single example from a tree simulated under the scenario of random auto-correlated changes of rate moving towards the tips, strongly biased towards a deceleration of evolutionary rates as time progresses (from the set of simulations used for Table 3). The true age of the root was 100 mya. Figure B shows the results using the MVS tree derived from the mitochondrial sequences of placental mammals. The dotted line indicates the 95% confidence interval of the estimated age of the root using the sum-of-squares approach described in Materials and Methods.

We begin the discussion by examining why the current cost functions, F_LOG_ and F_ADD_, overestimated the age of the root in the worked example with strong rate heterogeneity in the form of a short term highly elevated evolutionary rate. It is also important to examine the profile of the cost function around their minimal values for the age of the root. The functions F_LOG_ and F_ADD_ showed asymmetric behavior around the estimated root time, even in worked examples with a perfect molecular clock (data not shown), whereas the F_IR_ profile was symmetric and parabolic in shape. The asymmetry seen with F_LOG_ and F_ADD_ increased in response to a general bias towards deceleration of rates through time (Figure 7A). In particular, the function F_ADD_ showed a monotonic decrease with respect to increasing age of the root, that is, its estimate tended to infinity. Thus, asymmetric behavior of a cost function seems to be a symptom of unstable estimation of the age of the root. A similar strong asymmetry appeared in the profile of the current cost function with respect to the age of the root of the mitochondrial tree of placental mammals (Figure 7B), irrespective of whether MVS or ML branch lengths were used. Comparing Figures 7A and 7B suggests that bias due to decelerating evolutionary rates occurred in the evolutionary history of placental mammals, and is impacting the ability of current methods to estimate the correct age of the root.

Other approaches to divergence time estimation are under active development. For example, Drummond et al. [39] recently developed a set of Bayesian procedures to jointly estimate divergence times, evolutionary rates, and the tree topology. The uncorrelated variable rate model they use, which assumes independence of evolutionary rates between branches, may also be able to identify instantaneous accelerations. However, the magnitude of rate acceleration may be underestimated because hierarchical Bayes estimates can generally be regarded as shrinkage estimators [40]. An important future direction is to assess the performance of the various estimators in simulations where rate variability is a mixture of an autocorrelated process plus rare, but strong, instantaneous accelerations.

Our refined approaches resolve apparent contradictions between the quantitative molecular and paleontological data of placental mammals. Given such agreement, there is no need for ancillary hypotheses such as the long fuse model [2], [5], [9], [12]–[15], [41] to explain the lack of any positively identified fossils of modern placental mammals prior to about 75 million years ago. In addition, dates of markedly less than 100 mya for the root of placental mammals also bring into question earlier hypotheses that traditional continental drift models explain the geographic distribution of the four major groups of placental mammals [2]–[5], [13], [42]. We anticipate that the F_IR_ cost function developed in this paper will provide an improved methodology for a wide range of molecular studies because its robustness to rapid fluctuations of rate is essential to understanding events such as adaptive evolution. For example, because acquisition of new molecular functions can be achieved in a few million years after gene duplications [40], the duration of an inflated evolutionary rate may be surprisingly short, and, correspondingly, difficult to detect. We hope our new methods will be beneficial in such situations and will lead to a richer understanding of molecular evolution.

## Materials and Methods

### Autocorrelated variation of inverse rate

Because the branch lengths of a phylogenetic tree are estimated from the data as the product of the evolutionary rate of a branch and its time duration, these two factors cannot be directly estimated separately. It has become standard to use loose constraints to accommodate uncertainty and it is often wise to exclude constraints if there is no firm basis for them. Information on the age of some internal nodes may be fairly directly available from the fossil record. An example of this is the horse-rhino split [13]. If the molecular clock [16] governs the process of molecular evolution well, we can accurately estimate the times at other nodes given just a single reliable calibration point. However, the assumption of the molecular clock is often rejected by the fit of a clock-like tree to the data.

The most widely used assumption in order to model the evolutionary rate changes away form being constant (or away from a clock) is to introduce a stochastic process. For example, Sanderson [17] suggested the following cost function be minimized:
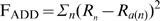
1with rates *R_n_* and *R_a_*_(*n*)_ being from the *n*th branch and its ancestral branch *a*(*n*), respectively. The exponent of the negative cost function Π*_n_* {(2π)^1/2^σ}^−1^ exp{−(*R_n_*−*R_a_*_(*n*)_)^2^/2σ^2^} implies that this function can be interpreted assumes *a priori* that rate changes follow a random walk with independent increments randomly drawn from a normal distribution. Likewise, Thorne et al. [31] considered a cost function of the type
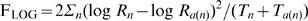
2in the framework of the hierarchical Bayes procedure. Here *T_n_* is the time interval of the *n*th branch (the current version of Multidivtime [31] uses rates at the nodes, instead of average rates along branches, as the free parameters). This can be interpreted as an assumption that the log rate undergoes Brownian motion. That is, independent increments of normal random variables, whose variances are proportional to the average time duration between the pair of branches (that is, the average length in time of the two branches). Recently, Sanderson implemented the unweighted cost function
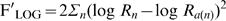
3into his program *r8s* [18].

However, the estimated rates based on any of the above cost functions may overly smooth the change of evolutionary rates when a pronounced transient change of rate has occurred. In turn, biased estimates of evolutionary rates will lead to biased estimates of divergence times. Here we propose a new cost function, which penalizes the local rate deviation from the harmonic mean. For simplicity, we ignore the stochastic variance of branch length [17], [18], [31], which goes to zero with increasingly long sequences. Denote the branch length and inverse rate of the *n*th branch by *B_n_* and *Q_n_* ( = *T_n_*/*B_n_*), respectively. The local rate variability (F_IR,*n*_) of two successive branches can be expressed as the variance of *Q_n_* and *Q_a_*_(*n*)_ around their average value, *A_n_* = (*Q_n_B_n_*+*Q_a_*_(*n*)_ *B_a_*_(*n*)_)/(*B_n_*+*B_a_*_(*n*)_), which is equal to the inverse of the harmonic mean of *R_n_* ( = *B_n_*/*T_n_*) and *R_a_*_(*n*)_, with the weights *B_n_* and *B_a_*_(*n*)_. That is, F_IR,*n*_ = {(*Q_n_*−*A_n_*)^2^ *B_n_*+(*Q_a_*_(*n*)_−*A_n_*)^2^ *B_a_*_(*n*)_}/(*B_n_*+*B_a_*_(*n*)_). Because F_IR,*n*_ is rewritten as F_IR,*n*_ = (*Q_n_*−*Q_a_*_(*n*)_)^2^ *W_n_* with the weight *W_n_* = *B_n_B_a_*_(*n*)_/(*B_n_*+*B_a_*_(*n*)_)^2^, the inverse-rate cost function, F_IR_, is defined as the weighted average of (*Q_n_*−*Q_a_*_(*n*)_)^2^ over all ancestor–descendant branch pairs in the tree:
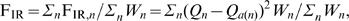
4which contrasts with previous expressions in terms of *R_n_*.

Because F_IR_ places a smaller penalty on abrupt rate changes than do previous models, it can confine an abrupt change close to where it occurred on the tree. In contrast, F_ADD_ and F_LOG_ *a priori* put stress upon the smoothness of evolutionary rate change from one branch to the next, and this may propagate the effect of an abrupt rate change over successive branches. In this article, the variable (*T_n_*) (the divergence times) were estimated by minimizing the cost functions (equations 1–4) subject to the (fossil) constraints without introducing other modifiers such as cross validation.

At first glance, it seems most intuitive to use a penalty such as (rate_1_−rate_2_)^2^; this has been the implicit assumption until now [31] and is the basis of published programs such as *r8s* and *Multidivtime* [17], [18], [31]. It rests on the implicit expectation that any reasonable type of smoothing of changes in rate will give a similar answer. However, a simple linear form for the difference in rates is misleading, and this is illustrated by considering the general problem of estimating velocity (rate) given distance (in our case branch length). Going from point *a* to *b* at velocity *v*_1_, then back at velocity *v*_2_, the average velocity (*v*_av_) is equal not to the standard arithmetic mean, but to the harmonic mean, 1/*v*_av_ = (1/*v*_1_+1/*v*_2_)/2. Further, going from point (node) *a* to *b* at velocity (rate) *v*_1_, and from point *b* to *c* at velocity *v*_2_, then the distances traveled (*d_ab_* and *d_bc_*) need no longer be equal so we must use weights. Specifically, *v*_av_ = (*d_ab_*+*d_bc_*)/(*t*_1_+*t*_2_), where *t*_1_ = *d_ab_*/*v*_1_ and *t*_2_ = *d_bc_*/*v*_2_. As a result, 1/*v*_av_ = (*d_ab_*/*v*_1_+*d_bc_*/*v*_2_)/(*d_ab_*+*d_bc_*). This is the same form as the average quantity *A_n_* used to obtain *F*_IR_ (equation 4). The use of the harmonic mean becomes important when *v*_1_ differs greatly from *v*_2_.

### The penalty in hierarchical Bayes and penalized likelihood approaches

The Bayesian approach estimates the divergence times and evolutionary rates in the form of a posterior distribution, which is summarized approximately as *P(* ***B*** *|* ***R*** *,* ***T*** *) exp(−λF)*. Here ***B***, R, and ***T*** are the vectors of the estimated branch lengths, evolutionary rates, and divergence times, respectively, while *F* is the cost function. The value of *λ* expresses the weight for the penalty, and is called a hyperparameter. Introducing the distribution for the hyperparameter is done via a so-called hyper-prior; the hyperparameter is then estimated concurrently with the posterior distribution. The penalized likelihood approach maximizes log *P(* ***B*** *|* ***R*** *,* ***T*** *)*−*λF*. The weight for the penalty *λ* is estimated using cross-validation. When the sequences are long enough, for example concatenated mitochondrial protein sequences, it is often safe to assume that the branch lengths are estimated accurately, that is with minimal stochastic error (but not necessarily without systematic error, something we address in this paper using MVS). Accordingly, it is assumed that the products of rates and times are known exactly. Thus in a situation of long, or very long sequences (e.g. genomic alignments), the results returned by using just the penalty function will converge to those returned by either a Bayesian or a penalized likelihood approach with the same type of cost function.

### MVS core-set correction of biased evolutionary distances

In the MVS method, the additivity of evolutionary distances is converted using the expected orthogonality among branch vectors in a multidimensional Euclidean space [25]. If the estimated pairwise distances do not satisfy additivity, MVS provides an index to measure deviations from orthogonality without specifying a tree structure. This index enables one to diagnose whether a distance matrix is compatible with a tree and to modify the biased distances until they satisfy orthogonality, that is, with a zero value for the index. Here, a revised core-set approach, that is, an extension of [27], is applied to correct the observed pairwise distances for convergent evolution.

The first step of this approach is to divide the set of taxa into subgroups and to correct the distances between pairs in each subgroup. We decompose the taxa based on partitions with both strong biological support and high bootstrap support. The deviation from additivity of pairwise distances is much smaller within each subgroup than across the whole distance matrix. If an anomalously large deviation is observed associated with a single taxon within a subgroup, that taxon is removed temporarily from the analysis. When the only deviations between distances within a subset of taxa are judged to be due to stochastic noise, the pairwise distances are modified by solving the equation of motion (a method using a many body kinetic equation in physics), which uses the index of the deviation from additivity as the potential energy [25], [26]. The modified distance matrix within a subgroup is interpreted as the true additive distance matrix (core set) achieved by minimal modification of the original distances. Generally, the modified distances are very close to the distances estimated by the NJ method for just these taxa. Then, the excluded taxa are deposited into this core set tree by solving the equation of motion with the same potential function. Assuming that convergent evolution and long-branch attraction are the main source of deviation from additivity, we allow for only positive corrections of the biased distances between the core set and excluded taxa (that is, distances can only get bigger).

The second step is a clustering of subgroups. The pairwise distances between two core-set groups are corrected by assuming attractions between them (that is, some distances are underestimated) and by minimal enlargement of the distances between the two groups to satisfy additivity. In this way, the core-set approach used in this article is in the direction opposite to that used in an earlier procedure that proceeds from the whole tree down to subtrees [26]. This revised method seems more appropriate here because the branching pattern within subgroups becomes more apparent and there are smaller deviations from additivity.

Conceptually, the first step of MVS core set is to decompose the whole tree into putative monophyletic groups that show minimal bias internally. The approach developed as part of this particular study resembles *k*-means clustering, that is, minimizing the deviation (variance) within groups while maximizing the deviation (variance) between groups. At present, the decomposition is done in a supervised, as opposed to an automatic, manner. In the second step, and when it is difficult to connect two groups definitely, we first exclude taxa with the largest deviations from additivity and then complete a new core set for the remaining sequences. We then locate the excluded sequences on the core set tree using minimal modification of distances (a parsimony-like criterion) under the constraint of only enlarging the distances identified as being biased (thus the only biases are assumed to be due to attraction).

When analyzing the mitochondrial protein sequences, we estimated pairwise distances initially under the JTT+Gamma model with α = 0.5. Given moderately high bootstrap support, the placental tree was decomposed into four major groups: Laurasiatheria, Supraprimate, Xenarthra, and Afrotheria. Laurasiatheria was decomposed into five subgroups, namely Cetartiodactyla, Perissodactyla, Carnivora, Chiroptera, and Euliptotyphyla. Supraprimates was decomposed into two subgroups: Primates and Glires. The core-set analysis began by generating additive distances within these subgroups, after which the clustering procedure described above was applied to connect these subgroups. In this analysis, we analyzed sequences of Xenarthra and Afrotheria together in a single core-set. This is because a relatively short internal branch separates them and Xenarthra contains but a single sequence in this analysis. Finally, we tried to modify the distances between placental mammals and the outgroups, but we could not determine the position of the root with confidence because of very strong attractions between many placental mammals and the outgroups. The consensus [3]–[5], [15] has been that the most probable placement of the root separates Afrotheria from the other placental mammals. The most likely alternative placement for the root is between Xenathra plus Afrotheria (Atlantogenata [2], [20]) and all other placentals (Boreotheria). Recent analyses including LINE sequences and indels [43] support this view. When we place the root in this position, the divergence times, including that of the root, change little (results not shown).

Further details of the current MVS procedure of distance modification are documented in Methods S1. The derived additive distance matrix was converted to a Newick tree file with the help of the NJ method, and this was used as the input data for divergence time estimation by the cost functions.

### Log-likelihood profiles of the cost function with respect to the age of the root

We note that least-squares estimators can be interpreted as ML estimators when errors in the data follow a multi-normal distribution. Accordingly, the least-squares residual can be treated as twice the log-likelihood ratio for the three cost functions G*_J_*(*T*_root_) (*J* = IR, ADD, or LOG); that is, as G*_J_*(*T*_root_) = *N* log{F*_J_*(*T*_root_)/F*_J_*(*T_M_*)}. Here, F*_J_*(*T*_root_) gives the minimum residual value with all internal node times reestimated, except for the root time *T*_root_, and it becomes truly minimal at *T*_root_ = *T_M_*. Here, *N* is the number of terms in the cost function, which is equal to 2*t*−4, where *t* is the number of tips on the tree (here *t* = 62, so *N* = 120). The 95% CI can be obtained using the threshold of G*_J_*(*T*_root_) = 3.84. Here, the minimum for criterion F_ADD_ was arbitrarily set to F_ADD_(*T*_root_) at *T*_root_ = 200 because the profile decreased monotonically for all greater values of the root time. In all evaluations, we avoid putting arbitrary constraints near the root of the tree (for example, that the rate at the root is the same as that of one of its descendants) because while arbitrary constraints may bound the minimum, they will also create unknown biases. Note that the MVS-F_IR_ profile is symmetric and parabolic around the estimated root time, whereas those of F_LOG_ and F_ADD_ are not. We see the same thing even in worked examples using a perfectly clock-like tree.

Our novel way of estimating CIs jointly takes into account some, but not all, of the sources of error noted in a previous publication [13]. These include errors in edge length estimation (because of a finite sampling of sites), the unpredictability of fluctuating evolutionary rates, and coalescent time. The assumptions of this method, vis-à-vis independence of errors, are the same as those used in programs by Thorne et al. [31]. Note that even if edge lengths have no error due to sampling of sites (e.g., the type of error identified by the bootstrap), they may still show four wide CIs because of the other two factors, which do not go to zero with increasing sequence length. This, in turn, shows that using the sequence bootstrap to infer errors on divergence times [17], [18] is not comprehensive and therefore the CIs are too narrow. We expect that this empirical approach to estimating standard errors will become increasingly accurate with increased numbers of internal nodes and calibration points.

Fortran source code and executable versions of the programs used in this study are downloadable from 〈http://www.kochi-ms.ac.jp/∼ct_cmis/kitazoe/〉. The operation of these programs, using the data in this analysis, is documented in Methods S2.

## Supporting Information

Methods S1 The procedure of MVS distance modification for placental mammals
(0.04 MB PDF)

Methods S2 The operation manual of programs for divergence time and MVS analyses
(0.04 MB PDF)

Figure S1 Trace of evolutionary rates along seven lineages in the MVS-F_IR_ and ML-F_IR_ analyses. The ML-F_IR_ analysis (B) showed a flatter peak rate than did the MVS-F_IR_ tree (A) (the black lines of F in the whale lineage) and inferred a longer period at a lower rate of evolution, producing an older root time. If lineages appear to merge with each other, zoom in to follow their exact path.
(0.68 MB TIF)

Table S1 Sequences and accession numbers used in this paper. The aligned amino acid sequences of the mitochondrial proteins ND1-ND6, ND4L, CO1-CO3, CTYB, and ATP6, are concatenated giving a total length of 3660 sites.
(0.09 MB DOC)

Table S2 Divergence times of the MVS-F_IR_, ML-F_IR_, and ML-F_LOG_ analyses. The numbers 1–61 denote the ancestral nodes in Figure 1. The red numbers stand for the positions of the time constraints. Times older than the constraints varied the most, particularly using ML branch lengths.
(0.09 MB DOC)
