# Supplementary material for: Robust Time Estimation Reconciles Views of the Antiquity of Placental Mammals
Source: PLoS One. 2007 Apr 18;2(4):e384. doi: 10.1371/journal.pone.0000384 (PMC1849890; doi:10.1371/journal.pone.0000384)
Supplement: Methods S1 — The procedure of MVS distance modification for placental mammals (0.04 MB PDF) [file pone.0000384.s001.pdf]

Methods S1: The procedure of MVS distance modification

We document the procedure how the MVS tree was constructed by the core set approach. We have four commands of **AMINODIST**, **MVS-A**, **MVS-B** and **MVS-C** which can be executed in a DOSV prompt screen of the PROG.ZIP (the file name downloaded). **AMINODIST** estimates the pairwise distances among 69 mitochondrial sequences (stored as the MITSEQ file in PROG.ZIP), and generate the DMATRIX file (saved as the DISTM). To execute **MVS-A**, **MVS-B** and **MVS-C**, we need a "name file" (NAME**) to specify the taxa to be analyzed and the distance matrix files (DISTM**), as the input data. These three commands generate the distance matrix files, DMATA1, DMATB1 and DMATC1, respectively. The README file explains the details of operation with help of simple sample data more easily. All programs and data files cited in the following procedure are stored in PROG.ZIP.

1) The additive distance matrix of Afrotheria+Xenarthra group with 9 taxa: The command **MVS-A** formed a core set (additive distance matrix) for the sequences with small deviations from additivity by excluding three taxa of Hyrax, Aardvark and Golden mole with especially large deviations, and constructed a core set for these sequences so as to satisfy additivity, and generated the additive distance matrix (DISTMG1) of the whole group by depositing the three taxa on the core set. Here, the input files, NAMEM1 and DISTM, were used.

2) The additive distance matrix of Supraprimates group consisting of Primates (12 taxa) and Rodentia (9 taxa): **a)** The command **MVS-A** first formed a core set of Primates by excluding three taxa of Capuchin, Tree shrew and Baboon, and generated the additive distance matrix (DISTMG2A) by depositing these three taxa on the core set. Here, the input files, NAMEM2A and DISTM, were used. **b)** The command **MVS-A** formed a core set of Rodentia by excluding two taxa of Vole and Rabbit, and generated the additive distance matrix (DISTMG2B) by depositing them on the core set. Here, the input files, NAMEM2B and DISTM, were used. **c)** The command **MVS-B** derived the additive distance matrix (DISTMG2) of Supraprimates from the input files, NAMEM2, DISTM, DISTMG2A and DISTMG2B.

3) The additive distance matrix (DISTMG3) of Laurasiatheria with 32 taxa was also derived by using the commands **MVS-A** and **MVS-B.**

4) The connection of Supraprimates and Laurasiatheria: The command **MVS-B** to connect these two large groups formed a core set (DISTMG23A) of this group by excluding three sister-group of Hedgehog, L. e. hedgehog and Greater moonrat. Here, the input files, NAMEM23, DISTM, DISTMG2 and DISTMG3, were used. It next generated the additive distance matrix (DISTMG23) of Supraprimates, by connecting the sister-group (DISTMG3A picked up from DISTMG3) to the core set (DISTMG23A). Here, the input files, NAMEM23, DISTM, DISTMG23A and DISTMG3A were used.

5) The MVS distances of placental mammals: The command **MVS-B** connected generated the additive distance matrix (DISTMG123) of placental mammals. Here, the input files, NAMEM123, DISTM, DISTMG1 and DISTMG23 were used.

6) The connection of outgroup (7 taxa) and placental mammals: After we obtained the distance matrix (DISTMG4) for outgroup by using the command **MVS-A**, we tried to connect the two groups by the command **MVS-B** using the input files, NAMEM1234, DISTM, DISTMG123 and DISTMG4. However, it was difficult to determine the root position uniquely because of very strong attractions between outgroup and many of placental mammals. There appeared the choice of the ancestral node of placental mammals whether the tree should be separated into the two lineages of (Afrotheria+Xenarthra) and the others by this node, or it should be separated into those of Afrotheria and the others. We calculated these two cases by using the command **MVS-C** which can specify the separation pattern with use of the name files, NAMEM1234A and NAMEM1234B. The file, DISTMVS, gives the final additive distance matrix of the former case in which the input files, NAMEM1234A, DISTM, DISTMG123 and DISTMG4, were used.

7) The divergence time estimation: The distance matrix DISTMVS was converted to the Newick format, TIMEMVS.TRE (given by the command **NEIGHBOR** of the NJ method [38]). The command **DTIME** estimated the divergence times for three cost functions, F_I, F_L and F_R by using the input files of NAMEMAM, TIMEMVS.TRE and CALIBMAM for fossil constraints, and generated the clock-like tree file, DTIME (renamed as TIMEMVS) and the rate file, RATE, which traces the rate changes along the evolutionary pathway.
