# Supplementary material for: Robust Time Estimation Reconciles Views of the Antiquity of Placental Mammals
Source: PLoS One. 2007 Apr 18;2(4):e384. doi: 10.1371/journal.pone.0000384 (PMC1849890; doi:10.1371/journal.pone.0000384)
Supplement: Methods S2 — The operation manual of programs for divergence time and MVS analyses (0.04 MB PDF) [file pone.0000384.s002.pdf]

# Methods S2: Operation manual of divergence time and MVS analysis

All FORTRAN programs are executable with sample files which can be easily operated by entering commands in a DOSV prompt screen of the folder (PROG.ZIP) downloaded from http://www.kochi-ms.ac.jp/~ct_cmis/kitazoe/. Here, the names of available sample files appear in each screen of operation, as demonstrations. New files may be made in the same formats as them.

**A. Divergence time analysis**

We explain by using a simulated result of stochastic rate fluctuations with the symmetric tree with 32 terminals (the tree without the abrupt rate change in Figure 4A)

Three input files are needed for this analysis:

1) The ID numbers of taxa are given in the file saved as NAMEST1, whose data format is described in the end of this file.
2) A distance matrix is given in a file saved as TIMEST1 which has ".TRE" extension of the Newick format.
3) The condition of fossil constraints is given in the file saved as CALIBST1.

Entering the command **IRDIVTIME** in the DOS prompt and inputting the above-mentioned three files, successively, you can execute divergence time calculations using one of four cost functions, $F_{ADD}$, $F_{LOG}$, $F'_{LOG}$ and $F_{IR}$ (Equations (1-4) in the main text). **IRDIVTIME** generates the clock-like distance matrix, DTIME, and the rate file, RATE, which traces the rate changes along the evolutionary pathway. The tree file is formed by entering the command **NEIGHBOR** [38] (included in PROG.ZIP) with the input file, DTIME.

The files, NAMEST1, TIMEST2.TRE, and CALIBST1 are available for an evolutionary process with strong rate decelerations. The files, NAMEST1, TIMEST3.TRE, and CALIBST3, are available for an evolutionary process with an abrupt rate change. The files, NAMEMAM, TIMEMVS.TRE (MVS tree), TIMEML.TRE (ML tree), and CABIBMAM are available for placental mammal phylogeny.

**B. The MVS analysis**

We explain by using a simulated result of convergent evolution with the 25 terminal sequences (Figure 5).

Two kinds of input files are needed for the MVS analysis:

4) The first file is the name file including the ID numbers of taxa. Looking into the file, NAMESD, you find the data format described in the end of the file.

5) The second file includes a distance matrix. You may use the file, DISTS, which gives the initially estimated distance matrix.

Four executables are available in the DOSV prompt screen:

6) With the command **MVSMAP**, you find a description of how deviations from additivity are reduced by the step-by-step exclusion of taxa in the order of the largest deviations. The MVS tree structure in the x-y plane is shown by using EXCEL (Microsoft office). This command visually represents convergent evolutions among lineages.

**Example a):** After setting all ID numbers (1-25) in the second column of the NAMESD file to **1**, you enter **MVSMAP** in the DOSV prompt and then enter DISTS**.** Next, enter **1** twice. Then, a screen showing behaviors of deviations appears. Here, you enter **0** without any exclusion of taxa. Finally, you enter, for example, **5**, **24** and **25** as a triplet of probes for the MVS map. As a result, the obtained file, MAPPING, shows a clear separation of the four groups, (1-8), (9-16), (17-24) and outgroups (25), which is given by using EXCEL (Microsoft office).

7) With the command **MVSDEV**, you can examine a stability of tree structure by the step-by-step exclusion of taxa in the order of the largest deviations. A distance matrix except for taxa with large deviations is given.

8) With the command **MVS-A**, the pairwsie distances within a small group are modified to so as satisfy additivity.

**Example b):** After you defined the ID numbers of 1-8 in the second column of the name file, NAMESD, as **1** and the others as **0**, enter **MVS-A** in the

DOSV prompt. Then, first enter NAMESD in the prompt screen, and second DISTS**.** In this case, since you have a small value of deviations due, enter **0** as the number of taxon exclusion. The DMATB1 file is generated by this procedure (renamed as DISTSG1). Similarly, the ID numbers of (9-16) and (17-24) in NAMESD produce the DISTSG2 and DISTSG3 files, respectively.

9) With the command **MVS-B**, two groups with the internally additive distances (given by **MVS-A**) are connected: The initially estimated pairwsie distances between the two groups are modified so as to satisfy additivity.

**Example c):** To connect the two groups of 1 and 2 in the tree (Figure S1), you define the ID numbers (1-16) in the second column of the NAMESD file as **1**, and enter **MVS-B** in the DOSV prompt. You input NAMESD, DISTS, DISTSG1 and DISTSG2, successively. After looking at the behavior of deviations from the additivity, you finally enter **0**. Then, the DISTSG12 file is given by renaming the obtained DISTMATB1 file. By repeating this procedure with the DISTS, DISTSG12 and DISTSG3 files (you define the ID numbers (1-24) in the second column of the NAMESD file as **1**), you get the additive distance matrix file, DISTSG123, within the three groups 1-3.

**Example d):** To connect the outgroup (25) with the groups 1-3 in the tree (Figure S1), you define all ID numbers in the second column of NAMESD as **1**. After inputting NAMESD, DISTS, and DISTSG123 in the DOS prompt, successively, you enter **1** and then the ID number **25** since the outgroup include one sequence. You finally enter **0** for no-exclusion of taxa. The obtained DMATB1 file satisfies the additivity as a whole system, and is saved as the DISTSG1234 file. This final MVS tree, DISTSG1234, gives the branch lengths (red triangles) of Figure 2.

10) The command **MVS-C** has the same function as **MVS-B**. However, **MVS-C** should be used when it is not able to uniquely determine branches on which the common ancestral nodes of the two groups are located, and the branch positions have to be assumed.

**Example e):** you may consider connecting the two groups of 1 and 2 in the tree (Figure S1). In the third column of the NAMESD file, you have to set

the ID numbers (1-4) to **1**, the ID numbers (5-8) to **2**, the ID numbers (9-12) to **3**, and the ID numbers (12-16) to **4**.

- Once a distance matrix is given by the MVS, it is converted to a newwick format by using the command **NEIGHBOR**, and is used as the input file for **IRDIVTIME**.

**C. Calculation of pairwise distances among amino acid sequences**

The command **AMINODIST** calculates a distance matrix more precisely than previous modules. It can specify the alpha value of site heterogeneity and the four models of amino acid transitions. The mitochondrial amino acid sequence file, MITSEQ, is useful for a sample sequence data.

**We developed the programs by using FORTRAN language where a library of FIJUTSU was applied. If there are any questions, please inform Y. Kitazoe: kitazoey@kochi-u.ac.jp.**
