# Supplementary figures and images for: Robust Time Estimation Reconciles Views of the Antiquity of Placental Mammals
Source: PLoS One. 2007 Apr 18;2(4):e384. doi: 10.1371/journal.pone.0000384 (PMC1849890; doi:10.1371/journal.pone.0000384)

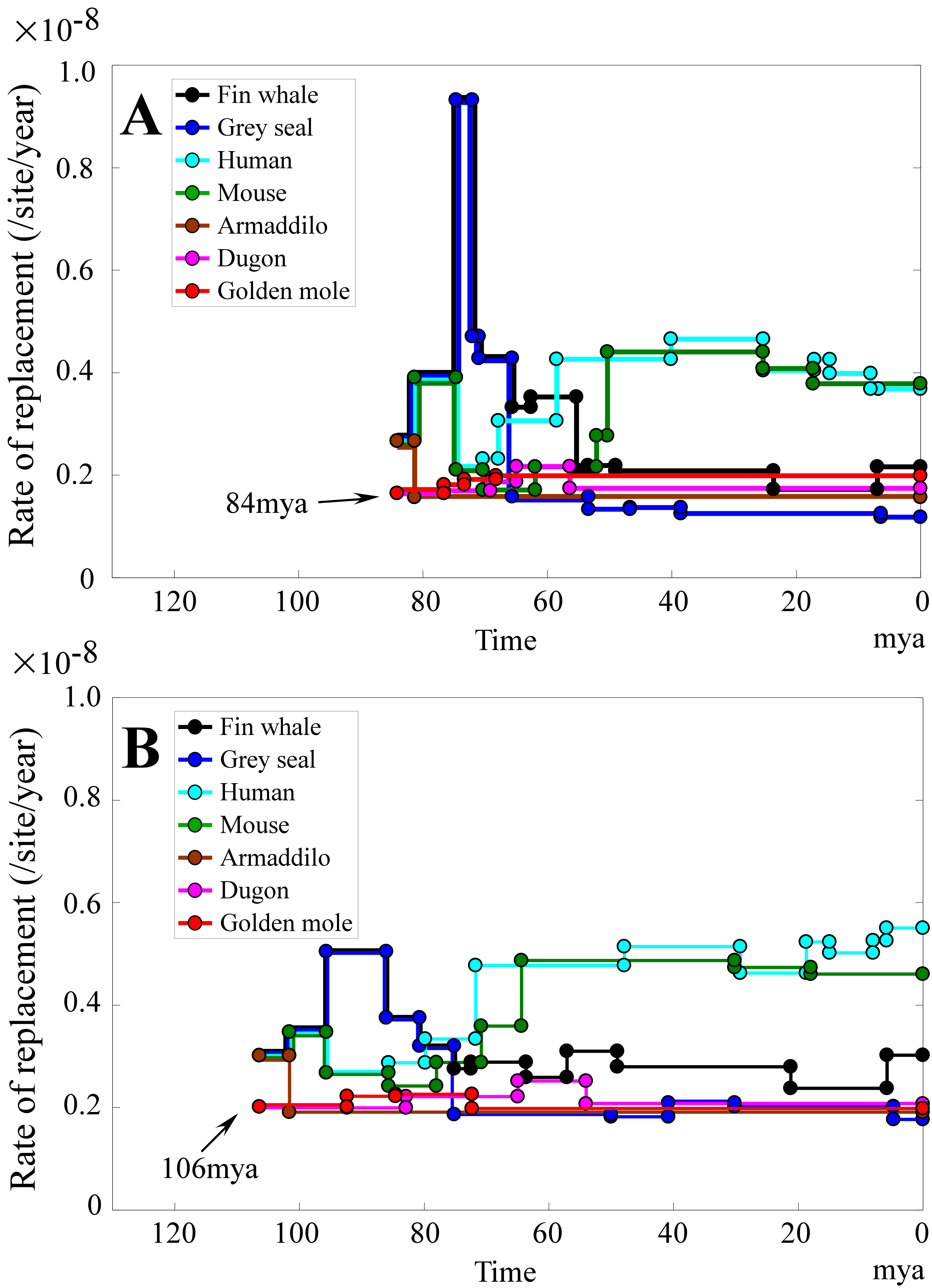

Supplement: Figure S1 — Trace of evolutionary rates along seven lineages in the MVS-FIR and ML-FIR analyses. The ML-FIR analysis (B) showed a flatter peak rate than did the MVS-FIR tree (A) (the black lines of F in the whale lineage) and inferred a longer period at a lower rate of evolution, producing an older root time. If lineages appear to merge with each other, zoom in to follow their exact path. (0.68 MB TIF) [file pone.0000384.s003.tif]
