# Supplementary material for: Robust Time Estimation Reconciles Views of the Antiquity of Placental Mammals
Source: PLoS One. 2007 Apr 18;2(4):e384. doi: 10.1371/journal.pone.0000384 (PMC1849890; doi:10.1371/journal.pone.0000384)
Supplement: Table S1 — Sequences and accession numbers used in this paper. The aligned amino acid sequences of the mitochondrial proteins ND1-ND6, ND4L, CO1-CO3, CTYB, and ATP6, are concatenated giving a total length of 3660 sites. (0.09 MB DOC) [file pone.0000384.s004.doc]

| **No.** |  | **Accession No.** | **Taxa name** |  | **No.** |  | **Accession No.** | **Taxa name** |
| --- | --- | --- | --- | --- | --- | --- | --- | --- |
| 1 |  | AF061340 | Jamaican fruit-eating bat |  | 36 |  | Y10524 | wallaroo |
| 2 |  | AB042770 | Ryukyu flying fox |  | 37 |  | X83427 | platypus |
| 3 |  | Y19192 | European mole |  | 38 |  | AF217811 | northern tree shrew |
| 4 |  | AJ002189 | pig |  | 39 |  | AJ238588 | Eurasian red squirrel |
| 5 |  | V00654 | cow |  | 40 |  | AB061526 | horseshoe bat |
| 6 |  | AF010406 | sheep |  | 41 |  | AB061528 | Japanese pipistrelle |
| 7 |  | AJ010957 | hippopotamus |  | 42 |  | AB061527 | long-clawed shrew |
| 8 |  | X61145 | fin whale |  | 43 |  | Y19184 | alpaca |
| 9 |  | X72204 | blue whale |  | 44 |  | AJ277029 | sperm whale |
| 10 |  | X63726 | harbor seal |  | 45 |  | AB099482 | Japanese mole |
| 11 |  | X72004 | grey seal |  | 46 |  | AB099483 | greater Japanese shrew-mole |
| 12 |  | U96639 | dog |  | 47 |  | AB099481 | long-eared hedgehog |
| 13 |  | U20753 | cat |  | 48 |  | AF348079 | greater moonrat |
| 14 |  | X79547 | horse |  | 49 |  | AF348081 | formosan shrew |
| 15 |  | X97337 | donkey |  | 50 |  | AF321050 | little red flying fox |
| 16 |  | X97336 | Indian rhinoceros |  | 51 |  | AF321051 | longtailed bat |
| 17 |  | Y07726 | white rhinoceros |  | 52 |  | AF406806 | formosan lesser horseshoe bat |
| 18 |  | Y11832 | nine-banded armadillo |  | 53 |  | AF303111 | polar bear |
| 19 |  | AJ224821 | African elephant |  | 54 |  | AJ309865 | barbary macaque |
| 20 |  | Y18475 | aardvark |  | 55 |  | AJ309866 | white-fronted capchin |
| 21 |  | AJ001588 | rabbit |  | 56 |  | AJ309867 | slow loris |
| 22 |  | AJ001562 | fat dormouse |  | 57 |  | AF348080 | American pika |
| 23 |  | AJ222767 | guinea pig |  | 58 |  | AJ301644 | greater cane rat |
| 24 |  | V00711 | mouse |  | 59 |  | AF348082 | vole |
| 25 |  | X14848 | rat |  | 60 |  | AJ421723 | dugong |
| 26 |  | D38112 | human |  | 61 |  | AJ400734 | tenrec1 |
| 27 |  | D38113 | chimpanzee |  | 62 |  | AB099484 | tenrec2 |
| 28 |  | D38116 | bonobo |  | 63 |  | AB096866 | cape golden mole |
| 29 |  | D38114 | gorilla |  | 64 |  | AB096867 | elephant shrew |
| 30 |  | D38115 | Bornean orangutan |  | 65 |  | AB096865 | cape hyrax |
| 31 |  | X97707 | Sumatran orangutan |  | 66 |  | AF357238 | silver-gray brushtail possum |
| 32 |  | X99256 | common gibbon |  | 67 |  | AF358864 | northern brown bandicoot |
| 33 |  | Y18001 | baboon |  | 68 |  | AJ304826 | common wombat |
| 34 |  | X88898 | hedgehog |  | 69 |  | AJ303116 | Australian echidna |
| 35 |  | Z29573 | North American opossum |  |  | | | |
