# Supplementary material for: Robust Time Estimation Reconciles Views of the Antiquity of Placental Mammals
Source: PLoS One. 2007 Apr 18;2(4):e384. doi: 10.1371/journal.pone.0000384 (PMC1849890; doi:10.1371/journal.pone.0000384)
Supplement: Table S2 — Divergence times of the MVS-FIR, ML-FIR, and ML-FLOG analyses. The numbers 1–61 denote the ancestral nodes in Figure 1. The red numbers stand for the positions of the time constraints. Times older than the constraints varied the most, particularly using ML branch lengths. (0.09 MB DOC) [file pone.0000384.s005.doc]

| **Node ID** | **MVS- F_IR_** | **ML- F_IR_** | **ML- F_LOG_** |  |  | **Node ID** | **MVS- F_IR_** | **ML- F_IR_** | **ML- F_LOG_** |  |
| --- | --- | --- | --- | --- | --- | --- | --- | --- | --- | --- |
| 1 | 6.9 | 5.7 | 6.8 |  |  | 32 | 24.2 | 22.4 | 25.5 |  |
| 2 | 23.6 | 21.2 | 23.1 |  |  | 33 | 17.4 | 17.0 | 18.1 |  |
| **3** | 49.0 | 49.0 | 49.0 |  |  | 34 | 54.4 | 57.3 | 58.9 |  |
| 4 | 53.5 | 57.1 | 57.4 |  |  | 35 | 3.4 | 2.8 | 3.7 |  |
| 5 | 55.3 | 63.7 | 63.7 |  |  | 36 | 6.7 | 5.8 | 7.6 |  |
| 6 | 62.6 | 72.5 | 73.5 |  |  | 37 | 8.0 | 8.0 | 10.4 |  |
| 7 | 65.7 | 75.2 | 76.5 |  |  | 38 | 14.6 | 14.9 | 19.0 |  |
| 8 | 71.0 | 80.8 | 83.8 |  |  | 39 | 17.1 | 18.7 | 23.5 |  |
| 9 | 72.0 | 86.0 | 91.7 |  |  | 40 | 25.3 | 29.3 | 35.2 |  |
| 10 | 74.7 | 95.7 | 108.5 |  |  | 41 | 40.1 | 47.9 | 55.5 |  |
| 11 | 81.3 | 101.6 | 116.4 |  |  | 42 | 58.5 | 71.7 | 82.7 |  |
| 12 | 84.2 | 106.4 | 122.2 |  |  | 43 | 67.9 | 79.8 | 91.5 |  |
| 13 | 28.2 | 28.8 | 30.5 |  |  | 44 | 70.4 | 85.7 | 97.7 |  |
| 14 | 53.1 | 58.4 | 58.6 |  |  | 45 | 4.5 | 4.3 | 5.5 |  |
| 15 | 10.2 | 9.5 | 10.6 |  |  | 46 | 10.4 | 11.7 | 14.3 |  |
| **16** | 52.0 | 52.0 | 53.1 |  |  | **47** | 17.3 | 18.0 | 19.9 |  |
| 17 | 29.4 | 24.4 | 25.2 |  |  | 48 | 25.3 | 30.2 | 33.7 |  |
| 18 | 6.4 | 4.7 | 5.2 |  |  | 49 | 50.3 | 64.4 | 74.3 |  |
| 19 | 38.5 | 30.2 | 32.8 |  |  | 50 | 52.1 | 70.8 | 81.7 |  |
| 20 | 46.7 | 40.8 | 43.8 |  |  | 51 | 62.0 | 78.0 | 89.4 |  |
| **21** | 53.8 | 50.0 | 52.8 |  |  | 52 | 40.2 | 47.2 | 53.9 |  |
| 22 | 10.9 | 11.0 | 11.4 |  |  | 53 | 44.5 | 55.5 | 65.0 |  |
| 23 | 50.2 | 52.2 | 52.1 |  |  | **54** | 55.0 | 55.0 | 55.0 |  |
| **24** | 55.8 | 60.0 | 60.0 |  |  | 55 | 56.4 | 54.1 | 54.1 |  |
| 25 | 60.4 | 67.0 | 67.5 |  |  | **56** | 65.0 | 65.0 | 65.0 |  |
| 26 | 1.6 | 1.9 | 2.1 |  |  | 57 | 69.2 | 82.9 | 88.9 |  |
| 27 | 26.5 | 23.1 | 22.6 |  |  | 58 | 76.7 | 92.4 | 102.3 |  |
| 28 | 18.3 | 18.1 | 20.8 |  |  | 59 | 2.3 | 2.3 | 2.5 |  |
| 29 | 30.3 | 32.3 | 36.5 |  |  | 60 | 73.4 | 84.6 | 92.9 |  |
| 30 | 60.4 | 66.9 | 73.0 |  |  | 61 | 68.2 | 72.3 | 78.8 |  |
| **31** | 69.4 | 80.2 | 85.4 |  |  |  |  |  |  |  |
